# JMJD6 negatively regulates cytosolic RNA induced antiviral signaling by recruiting RNF5 to promote activated IRF3 K48 ubiquitination

**DOI:** 10.1371/journal.ppat.1009366

**Published:** 2021-03-08

**Authors:** Wei Zhang, Qi Wang, Fan Yang, Zixiang Zhu, Yueyue Duan, Yang Yang, Weijun Cao, Keshan Zhang, Junwu Ma, Xiangtao Liu, Haixue Zheng

**Affiliations:** 1 State Key Laboratory of Veterinary Etiological Biology, National Foot and Mouth Diseases Reference Laboratory, Lanzhou Veterinary Research Institute, Chinese Academy of Agricultural Sciences, Lanzhou, China; 2 National Agricultural Science & Technology Center, Chinese Academy of Agricultural Sciences, Chengdu, China; University of Southern California, UNITED STATES

## Abstract

The negative regulation of antiviral immune responses is essential for the host to maintain homeostasis. Jumonji domain-containing protein 6 (JMJD6) was previously identified with a number of functions during RNA virus infection. Upon viral RNA recognition, retinoic acid-inducible gene-I-like receptors (RLRs) physically interact with the mitochondrial antiviral signaling protein (MAVS) and activate TANK-binding kinase 1 (TBK1) to induce type-I interferon (IFN-I) production. Here, JMJD6 was demonstrated to reduce type-I interferon (IFN-I) production in response to cytosolic poly (I:C) and RNA virus infections, including Sendai virus (SeV) and Vesicular stomatitis virus (VSV). Genetic inactivation of JMJD6 enhanced IFN-I production and impaired viral replication. Our unbiased proteomic screen demonstrated JMJD6 contributes to IRF3 K48 ubiquitination degradation in an RNF5-dependent manner. Mice with gene deletion of JMJD6 through piggyBac transposon-mediated gene transfer showed increased VSV-triggered IFN-I production and reduced susceptibility to the virus. These findings classify JMJD6 as a negative regulator of the host’s innate immune responses to cytosolic viral RNA.

## Author summary

RLRs-mediated signaling needs to be terminated in order to prevent persistent immune responses and adverse effects to the host once the virus has been cleared. In this study, we provide rigorous evidence that JMJD6 negatively regulates RLRs-mediated innate immune responses. We found that JMJD6 recruits RNF5 to induce the K48-linked polyubiquitination and proteasomal degradation of activated IRF3. Genetic inactivation of JMJD6 in cells increases IFN-I production to suppress viral infection. Consistently, *in vivo* studies show that, compared with WT mice, JMJD6-deficient mice are more resistant to VSV infection with more IFN-I production and reduced viral load in livers. Our findings reveal a novel mechanism to downregulate innate immune responses mediated by RNA viral infection, which allows the host to prevent undue immune responses and sustain homeostasis.

## Introduction

Innate immunity is the first line of host defense against viral infection [1]. Host utilizes pattern recognition receptors (PRRs) to sense pathogen-associated molecular patterns (PAMPs) [2]. For example, recognition of double-stranded RNA (dsRNA) during viral infections by cytosolic PRRs such as MDA5, RIG-I and MAVS, leads to the production of type I interferon (IFN-I) and hundreds of direct antiviral IFN-stimulated genes (ISGs) [3, 4]. IFN-I and ISGs are protective in viral infection and orchestrate adaptive antiviral immunity [5, 6], but excessive IFN-I production can have harmful roles for the host in certain chronic viral infections and several autoimmune diseases. [5]. Thus, a fundamental question for IFN-I signaling is: How does the host regulate the IFN-I production to modulate innate immune responses in a balanced manner?

IFN-I signaling is critical for eliminating invading viruses but sustained IFN-I production is detrimental to immune responses and homeostasis [7, 8]. Among the regulatory molecules of IFN-I, activated IRF3 is essential for the activation of the IFN-I promoter and subsequent IFN-I production [9]. IRF3 activation is well known to form phosphorylation-dependent dimerization upon viral infection [10]. Several studies have demonstrated that IRF3 activation is terminated by dephosphorylation and polyubiquitination. For example, RACK1 recruits phosphatase PP2A to dephosphorylate activated IRF3 [11]. The peptidyl-prolyl isomerase Pin1 interacts with phosphorylated IRF3 and eventually results in proteasome-dependent degradation of IRF3 [12]. TRIM26 bound to IRF3 and promoted its K48-linked polyubiquitination and degradation [13]. These studies demonstrate that IRF3 is under exquisite regulation, but the regulatory mechanisms remain incompletely defined.

JMJD6 is one of the Jumonji C domain-containing proteins (JMJD) family that composed of more than 10 members in humans [14]. JMJD proteins perform as epigenetic regulators to demethylate substrate proteins [15]. Tikhanovich et al. demonstrated that JMJD6 demethylated TRAF6 to regulate Toll-like receptor signaling [16]. Besides, JMJD6-induced demethylation of STAT1 suppresses the expression of ISGs by attenuating JAK-STAT signaling, resulting in enhanced HCV replication [17]. Here, we have established a novel role for JMJD6 as a negative regulator of the host’s innate immune response to cytosolic viral RNA. JMJD6 suppresses cytosolic viral RNA induced IFN-I production. JMJD6 recruits RNF5 to degrade activated IRF3 to maintain the immune homeostasis and prevent overwhelming innate immunity. Our findings indicate that JMJD6 is crucial for the control of IRF3 activation.

## Results

### JMJD6 attenuates the type I interferon production

**Fig 1:**
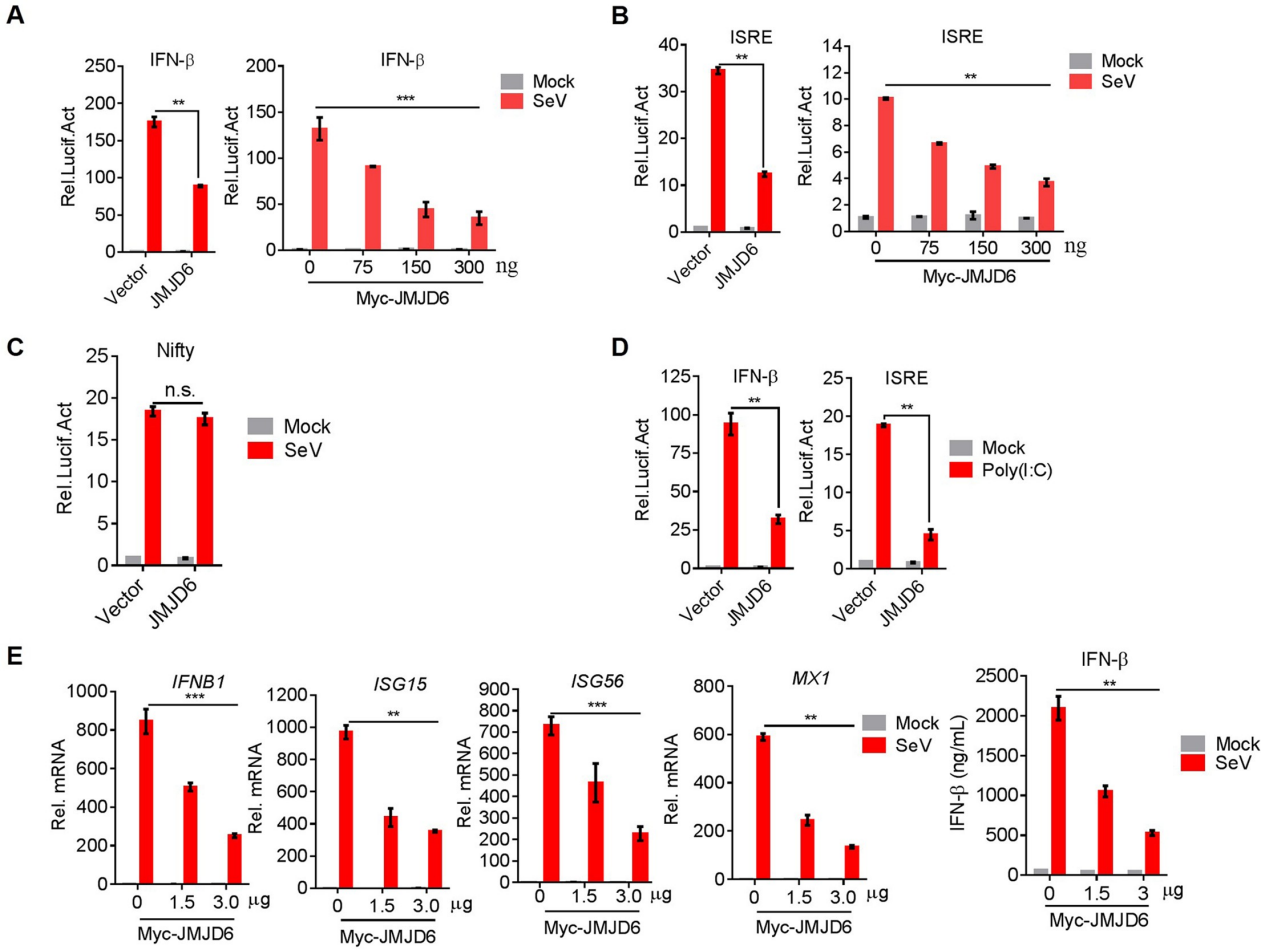
JMJD6 attenuates the type I interferon production. (A) JMJD6 suppresses SeV-induced transcriptional activation of the IFN-β promoter in a dose-dependent manner. HEK293T cells were transfected with Myc-JMJD6 or empty vector together with the IFN-β luciferase reporters. At 24 h post-transfection (hpt), cells were mock-infected or infected with SeV for 12 h, and luciferase assays were performed 12 h later using a dual-specific luciferase assay kit. Statistical significance was determined with a two-tailed Student’s t-test (*** *P*<0.001, **P<0.01, and *P<0.05). (B) JMJD6 inhibits SeV-induced transcriptional activation of the ISRE promoter in a dose-dependent manner. HEK293T cells were transfected with the indicated plasmids and then mock-infected or infected with SeV for 12 h. Luciferase assays were performed using a dual-specific luciferase assay kit. (C) The effect of JMJD6 on SeV-induced transcriptional activation of the Nifty promoter. (D) JMJD6 suppresses poly(I:C)-induced transcriptional activation of IFN-β promoter and ISRE. HEK293T cells were transfected with Myc-JMJD6 or empty vector together with the IFN-β or ISRE luciferase reporters. At 24 hpt, cells were transfected by lipofectamine 2000 with or without poly(I:C) (50 ng/mL) for 12 h. Luciferase assays were performed with a dual-specific luciferase assay kit. (E) JMJD6 inhibits the transcription and secret of IFN-β and the transcription of its downstream ISGs in a dose-dependent manner. The mRNA levels of IFN-β, ISG15, ISG56, and MX1 were detected by qPCR. The IFN-β production was detected by ELISA.

To determine the function of JMJD6 in the innate immunity, whether JMJD6 had a substantial impact on IFN-I production was investigated. We transfected HEK293T cells with an IFN-β promoter-driven luciferase reporter, internal control renilla luciferase reporter and vector encoding JMJD6 or empty vector. We found JMJD6 substantially reduced IFN-I production upon SeV infection in a dose-dependent manner (Fig 1A).

Overexpression of JMJD6 did not activate the IFN-β promoter-driven luciferase reporter, indicating JMJD6 is not an activator of IFN-I production (Fig 1A). IFN-stimulated response element (ISRE) and NF-κB were required for activation of IFN-β induction [18]. Therefore, we tested the effect of JMJD6 on an ISRE promoter-driven luciferase reporter or an NF-κB promoter-driven luciferase reporter. We found JMJD6 substantially reduced ISRE promoter-driven luciferase activities in a dose-dependent manner upon SeV infection (Fig 1B). But JMJD6 doesn’t affect NF-κB promoter-driven luciferase activities (Fig 1C). All of these indicate JMJD6 suppresses IFN-I production through NF-κB-independent pathways. VSV infection or cytosolic poly(I:C) can activate MDA5/RIG-I-MAVS-dependent IFN-I production [4]. We transfected HEK293T cells with an IFN-β or ISRE promoter-driven luciferase reporter and internal control Renilla luciferase reporter and vector encoding JMJD6 or empty vector upon cytosolic poly(I:C) activation. As shown in Fig 1D, JMJD6 substantially reduced IFN-β or ISRE promoter-driven luciferase activities, consistent with the effect of JMJD6 on SeV infection. As reporter assay is an artificial system, we tested endogenous induction of IFN-β and ISGs upon SeV infection in HEK293T cells. Like the IFN-β or ISRE promoter-driven luciferase reporter activation, JMJD6 substantially suppressed the production of IFN-β and ISGs, including ISG15, ISG56, and MX1 upon SeV infection (Fig 1E). Taken together, all of these data indicate JMJD6 suppresses the cytosolic RNA-induced IFN-I production.

### Deficient JMJD6 leads to potentiate the type I interferon production

**Fig 2:**
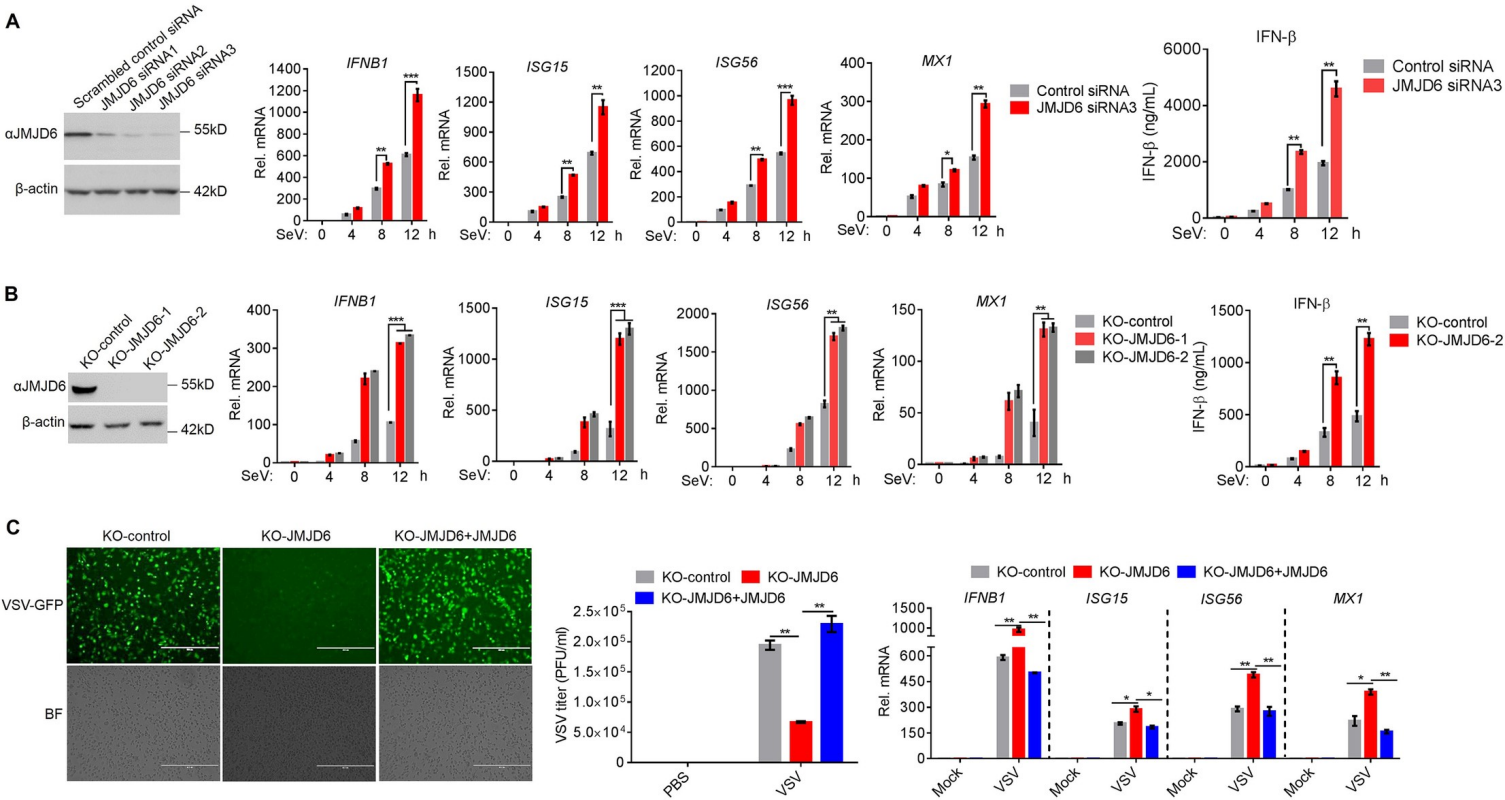
JMJD6 deficiency potentiates the type I interferon production. (A) Evaluation of the knockdown efficiency of JMJD6-siRNA. HEK293T cells were transfected with 150 nM NC or si-JMJD6, and the expression of the JMJD6 protein was detected by immunoblotting using an anti-JMJD6 antibody (Left panel). Effects of si-JMJD6 on SeV-stimulated induction of the expression of IFN-β and its known downstream ISG genes. The mRNA levels of IFN-β, ISG15, ISG56, and MX1 were detected by qPCR. The IFN-β production was detected by ELISA (Right panel). (B) Immunoblotting analysis of JMJD6 protein levels in KO-JMJD6 HeLa cells (Left panel). Effects of KO-JMJD6 on SeV-stimulated induction of the expression of IFN-β and its known downstream ISG genes (Right panel). (C) Effects of JMJD6 on the replication of vesicular stomatitis virus (VSV). KO-control cells, KO-JMJD6 cells, or KO-JMJD6 cells reconstituted with JMJD6 were infected with VSV-GFP (MOI = 0.01) for 24 h, and viral infection and titers were detected using fluorescence microscopy and plaque assay, and the ISG56, MX1, ISG15, and IFN-β mRNAs was detected by qPCR.

We next endeavored to determine the endogenous function of JMJD6 on IFN-I production. JMJD6 was knocked down in HEK293T cell by siRNAs before SeV infection. As shown in Fig 2A, three JMJD6 siRNA molecules were used to identify the most effective one. Of the three JMJD6 siRNAs, siRNA3 was the most effective, silencing JMJD6 expression by over 90% (Fig 2A). We opted to use JMJD6 siRNA3 in further experiments. As shown in Fig 2A, SeV infection leads to more expression of IFN-β and the ISGs, including ISG15, ISG56, and MX1 in HEK293T cells containing JMJD6 siRNA3, compared to the cells containing the scrambled control siRNA. ELISA results indicated that knockdown of JMJD6 expression enhanced IFN-β production (Fig 2A). To further confirm the role of JMJD6 in modulating cytosolic RNA-induced IFN-I production, we eliminated JMJD6 expression by CRISPR-Cas9 gene editing in HeLa cells (S1 Fig). JMJD6 expression was detected in HeLa cells transduced with a non-targeting single guide RNA (sgRNA) with a scrambled sequence (named as ’KO-control’) but not in either of two independent HeLa cell lines (KO-JMJD6-1 and KO-JMJD6-2) transduced with different JMJD6-specific sgRNA (Fig 2B). We infected the cells directly with SeV. We observed substantially higher mRNA expression of the IFN-β and the ISGs, including ISG15, ISG56, and MX1 in SeV-infected JMJD6-deficient (KO-JMJD6-1 and KO-JMJD6-2) cells than that in SeV-infected control HeLa cells, and ELISA showed similar results (Fig 2B). VSV-GFP was introduced here to infect both KO-control and KO-JMJD6 cells. As shown in Fig 2C, there were more GFP-positive populations and viral titers in KO-control cells than that in the KO-JMJD6 cells. And we consistently observed enhanced IFN-β, ISG15, ISG56, and MX1 mRNA expression in the KO-JMJD6 cells, compared to the KO-control cells. When JMJD6 was reconstituted into KO-JMJD6 cells, JMJD6 rescued viral infection compared with KO-JMJD6 cells (Fig 2C). Collectively, these results defined JMJD6 as a negative regulator of IFN-I production to RNA viruses infection.

### JMJD6 inhibits RIG-I-MAVS-TBK1 signaling via reducing IRF3 expression

**Fig 3:**
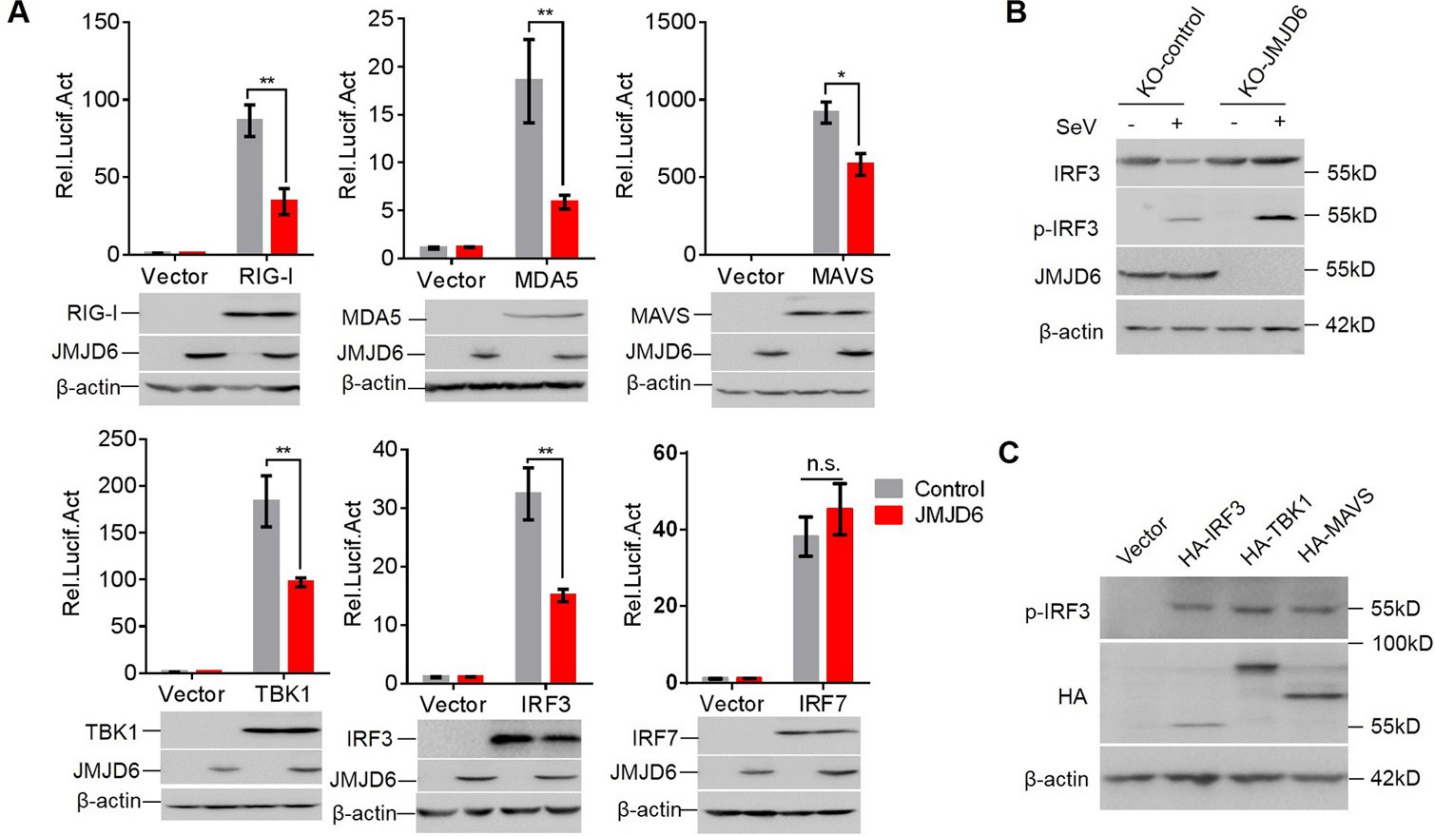
JMJD6 inhibits RIG-I-MAVS-TBK1 signaling via reducing IRF3 expression. (A) JMJD6 inhibits virus-induced IFN-β production at the IRF3 level. HEK293T cells were transfected with the indicated plasmids for 24 h. Luciferase assays were performed with a dual-specific luciferase assay kit. Protein expression was analyzed by Western blotting. (B) Endogenous JMJD6 reduces phosphorylated IRF3 (p-IRF3) and total IRF3. KO-control or KO-JMJD6 cell line was infected with or without SeV for 12 h. The p-IRF3, total IRF3, JMJD6, and β-actin were detected. (C) IRF3 is phosphorylated in the IRF3 overexpression situation. HEK293T cells were transfected with the indicated plasmids, and the expression of phosphorylated IRF3 was determined by immunoblot.

RIG-I-like receptors recognize cytosolic RNA and then activate MAVS. MAVS physically interacts with TBK1 to phosphorylate and activate IRF3 or IRF7 for IFN-I production [7, 19]. Overexpression of these components is well known to activate IFN-I production. To explore the mechanism by which JMJD6 reduced the RIG-I-mediated signaling cascade, we tested the effect of JMJD6 on IFN-β promoter-driven luciferase activities by these components. Overexpression of JMJD6 suppressed IFN-β promoter-reporter activation by RIG-I, MDA5, MAVS, TBK1, and IRF3 (Fig 3A). However, JMJD6 did not affect downstream IRF7-dependent IFN-β promoter-reporter activation (Fig 3A), indicating that JMJD6 targets the RIG-I-MAVS-TBK1 pathway at the node of IRF3. To dissect the role of JMJD6 on IRF3 activation, we examined the total and phosphorylated IRF3 upon SeV infection in KO-JMJD6 cells. As shown in Fig 3B, the total and phosphorylated IRF3 level was dramatically enhanced in KO-JMJD6 cells upon SeV infection. Of note, JMJD6 did not affect the basal IRF3 expression, suggesting JMJD6 reduces IRF3 expression in an activation-dependent manner. To test whether IRF3 was phosphorylated or not in the IRF3 overexpression situation, HEK293T cells were transfected with HA-IRF3, HA-TBK1, or HA-MAVS plasmids, the IRF3 phosphorylation was detected, as shown in Fig 3C, IRF3 was phosphorylated in the IRF3, TBK1, or MAVS overexpression, which is consistent with other studies [13].

### JMJD6 promotes activated IRF3 ubiquitination and degradation

**Fig 4:**
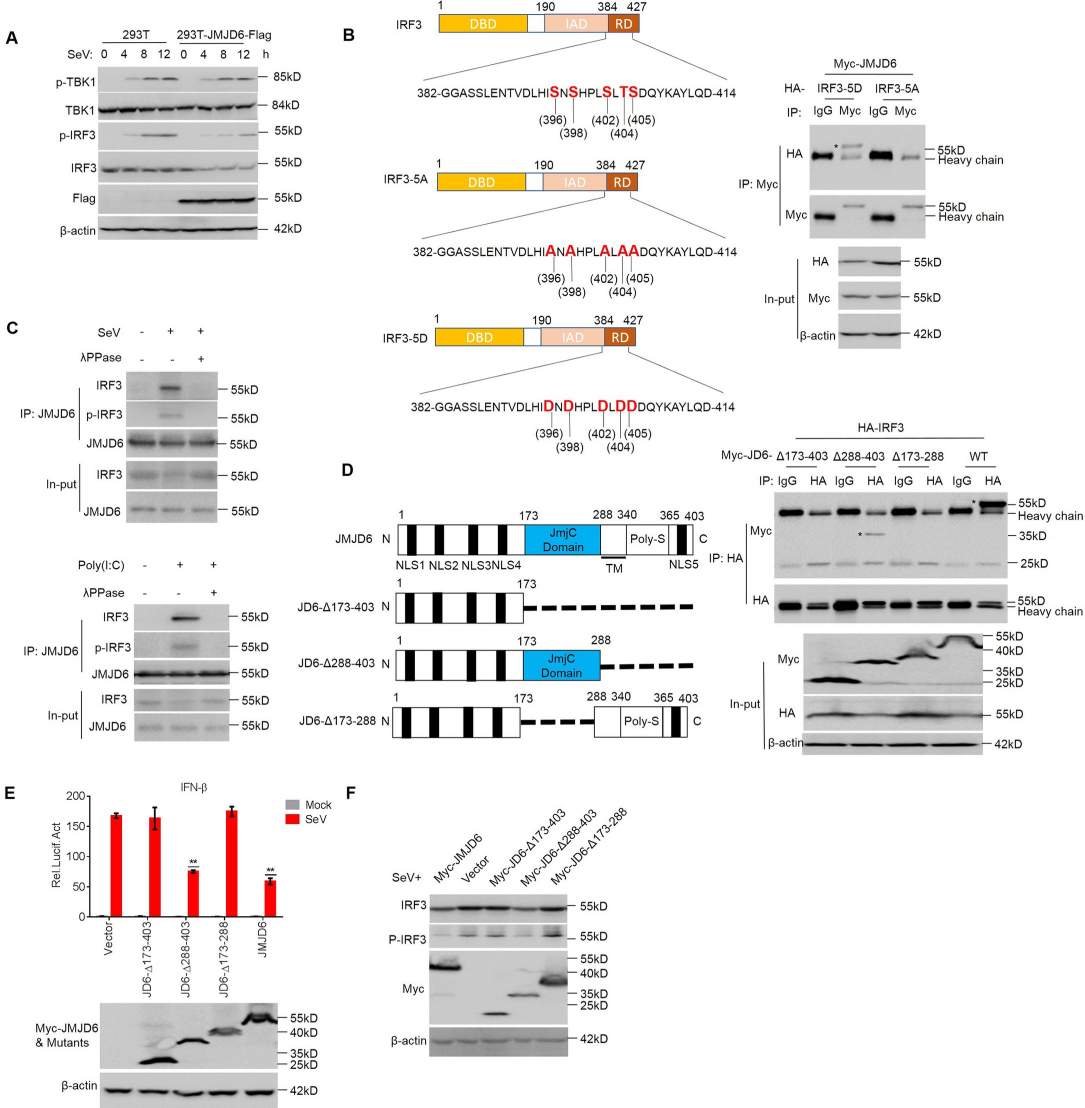
JMJD6 promotes activated IRF3 degradation. (A) HEK293T cells and HEK293T-JMJD6-Flag cell lines were infected with SeV as indicated, and the phosphorylation levels of IRF3 and TBK1 were analyzed. (B) Diagrams of IRF3 and its mutants. DBD, DNA binding domain; IAD, IRF3 association domain; RD, a C-terminal regulatory domain (left panel). HEK293T cells were transfected with the indicated plasmids, and cell lysates were immunoprecipitated with anti-Myc or IgG antibody followed by immunoblot using anti-HA and anti-Myc antibodies (right panel). (C) Endogenous JMJD6 interacts with IRF3 or p-IRF3 after a viral infection or poly(I:C) stimulation. HEK293T cells were infected with SeV for 12 h. Cells lysates were immunoprecipitated with anti-IRF3 or anti-p-IRF3, and the immunoprecipitates were analyzed by immunoblot with anti-JMJD6 antibody (top panel). Immunoblot of lysates from HEK293T cells stimulated with poly(I:C) for 12 h, analyzed with anti-IRF3 antibody (below panel). (D) Diagrams of JMJD6 and its mutants (Left panel). HEK293T cells transfected with the indicated plasmids and stimulated with poly(I:C) as indicated. The cell lysates were immunoprecipitated with an IgG or anti-HA antibody followed by immunoblots using anti-HA and anti-Myc antibodies. Asterisks represent target proteins (Right panel). (E) HEK293T cells were co-transfected with vector, Myc-JMJD6 or the JMJD6 mutants expressing plasmids and the IFN-β promoter-reporter plasmids for 24 h, and then infected with or without SeV for another 12 h. The luciferase activity was measured with a dual-luciferase assay. Expression of Myc-tagged JMJD6 protein and the mutant proteins was evaluated by Western blotting. (F) HEK293T cells were transfected with the indicated plasmids for 24 h and then infected with SeV for another 12 h. The phosphorylated IRF3 (p-IRF3), total IRF3, Myc-JMJD6 or mutants, and β-actin were detected by Western blotting.

To test whether JMJD6 uses additional mechanisms to target the IFN-I signaling pathway, we generated stable JMJD6-overexpressing HEK293T cell lines (HEK293T-JMJD6-Flag) by lentivirus infection and detected the phosphorylated TBK1, TBK1, phosphorylated IRF3, and IRF3 at different time point post-SeV infection (Fig 4A). IRF3 expression diminished with IRF3 phosphorylation at 4 h post-infection of SeV in JMJD6-overexpressing HEK293T cell lines (Fig 4A). To further confirm whether JMJD6 reduces IRF3 expression in an activation-dependent manner, we introduced a constitutively active IRF3 variant (IRF3-5D) in which Ser396, Ser398, Ser402, Thr404, and Ser405 were replaced by phosphomimetic aspartate (Fig 4B) [13, 20]. We also introduced the IRF3 variant (IRF3-5A) as a control, which is a phosphorylation deficient mutant (Fig 4B) [13]. Of note, we found JMJD6 interacts with IRF3-5D, but not the IRF3-5A (Fig 4B). These results are consistent with the result mentioned above that JMJD6 reduces IRF3 expression in an activation-dependent manner. Endogenous IRF3 can only interact with JMJD6 upon SeV infection and poly (I:C) activation because both SeV infection and poly (I:C) activation induced IRF3 activation (Fig 4C). Moreover, we utilized recombinant lambda protein phosphatase (λPPase) to dephosphorylate the proteins, and the results showed that λPPase treatment interrupted interaction between IRF3 and JMJD6 (Fig 4C). These results indicate IRF3 interacts with JMJD6 in an activation-dependent manner.

Reciprocal domain mapping experiments were conduced with JMJD6 deletion constructs and IRF3 upon poly (I:C) activation. The intermediate domain of JMJD6 (amino acids 173–288 of JMJD6) is essential for the interaction with IRF3 upon poly (I:C) activation (Fig 4D). Consistent with the interaction results, the amino acids 173–288 of JMJD6 is responsible for dramatically reducing IFN-I production, and the inhibition effect is very similar to that determined by full-length JMJD6 (Fig 4E). As shown in Fig 4F, the amino acids 173–288 of JMJD6 is sufficient to reduce activated IRF3.

**Fig 5:**
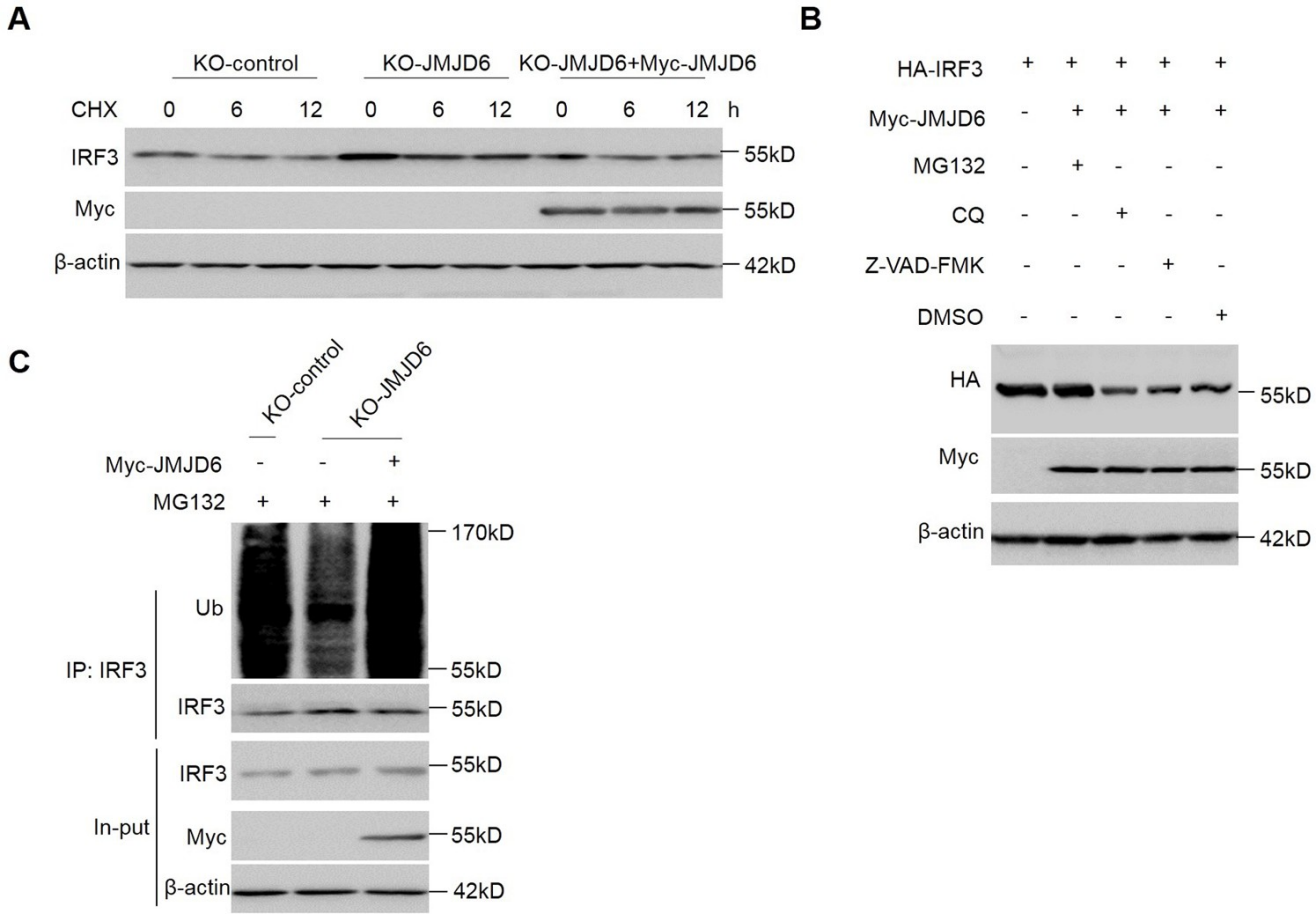
JMJD6 promotes activated IRF3 ubiquitination. (A) Endogenous JMJD6 regulates the stability of IRF3. KO-control cells, KO-JMJD6 cells, or KO-JMJD6 cells reconstituted with JMJD6 were infected with SeV for 6 h and then cultured in the presence of cycloheximide (CHX) for 0, 6, 12 h. Total IRF3, Myc-JMJD6, and β-actin were detected. (B) HEK293T cells were transfected with the indicated plasmids and then stimulated with poly(I:C) in the presence or absence of MG132 (20 μM), CQ (100 μM), or Z-VAD-FMK (50 μM) for 6 h. The expression of HA-IRF3 and Myc-JMJD6 proteins were detected by Western blotting. (C) Endogenous JMJD6 enhanced the ubiquitination of IRF3. KO-control cells, KO-JMJD6 cells, or KO-JMJD6 cells reconstituted with JMJD6 were infected with SeV for 6 h in the presence of MG132 (20 μM). Immunoblot analysis (with anti-Ub) of proteins immunoprecipitated (with anti-IRF3) was performed.

Inhibition of protein synthesis with cycloheximide (CHX) demonstrated that activated IRF3 is not a stable protein in cells with an approximate half-life of 6 h (Fig 5A and S2A Fig). IRF3 stability was examined using JMJD6-deficient and JMJD6-rescued cells upon virus infection. Increased stability of IRF3 in the absence of JMJD6 upon SeV infection and reconstitution of JMJD6 significantly counteracted increased stability of IRF3 in the JMJD6-deficient cells upon SeV infection (Fig 5A). Activated IRF3 half-life was reduced by JMJD6 overexpression in cells, but the deletion of the 173–288 functional domain of JMJD6 did not affect IRF3 half-life (S2A Fig). These indicate the amino acids 173–288 of JMJD6 is critical to the stability of IRF3 upon SeV infection.

In eukaryotes, ubiquitin-proteasome and lysosome-autophagy pathways are the two major systems for the degradation of cellular proteins to maintain homeostasis [21]. We treated cells with MG132, chloroquine (CQ), and pan-caspase inhibitor Z-VAD-FMK. As shown in Fig 5B, MG132 treatment effectively blocks activated IRF3 reduction in cells with overexpression JMJD6, which indicates JMJD6 promotes activated IRF3 degradation via the proteasomal pathway. Protein ubiquitination is a critical step in the proteasome degradation pathway. We determined IRF3 polyubiquitylation with overexpression JMJD6 or vector, as shown in S2B Fig, MG132 treatment accumulated polyubiquitylated IRF3 in cells with overexpression of JMJD6. Meanwhile, the IRF3 polyubiquitination was examined using JMJD6-deficient cells and JMJD6-rescued cells upon virus infection with MG132 treatment. Reduced polyubiquitination of IRF3 in the absence of JMJD6 upon SeV infection and reconstitution of JMJD6 significantly increased polyubiquitination of IRF3 in the JMJD6-deficient cells upon SeV infection (Fig 5C). In general, E3 ubiquitin ligases are essential in recognizing, binding, and covalently attaching ubiquitin to their substrates [22]. However, JMJD6 does not belong to E3 ubiquitin ligases; therefore, the mechanism of how JMJD6 promotes activated IRF3 degradation is elusive.

### JMJD6 promotes activated IRF3 K48 ubiquitination and degradation via ubiquitin ligase RNF5

**Fig 6:**
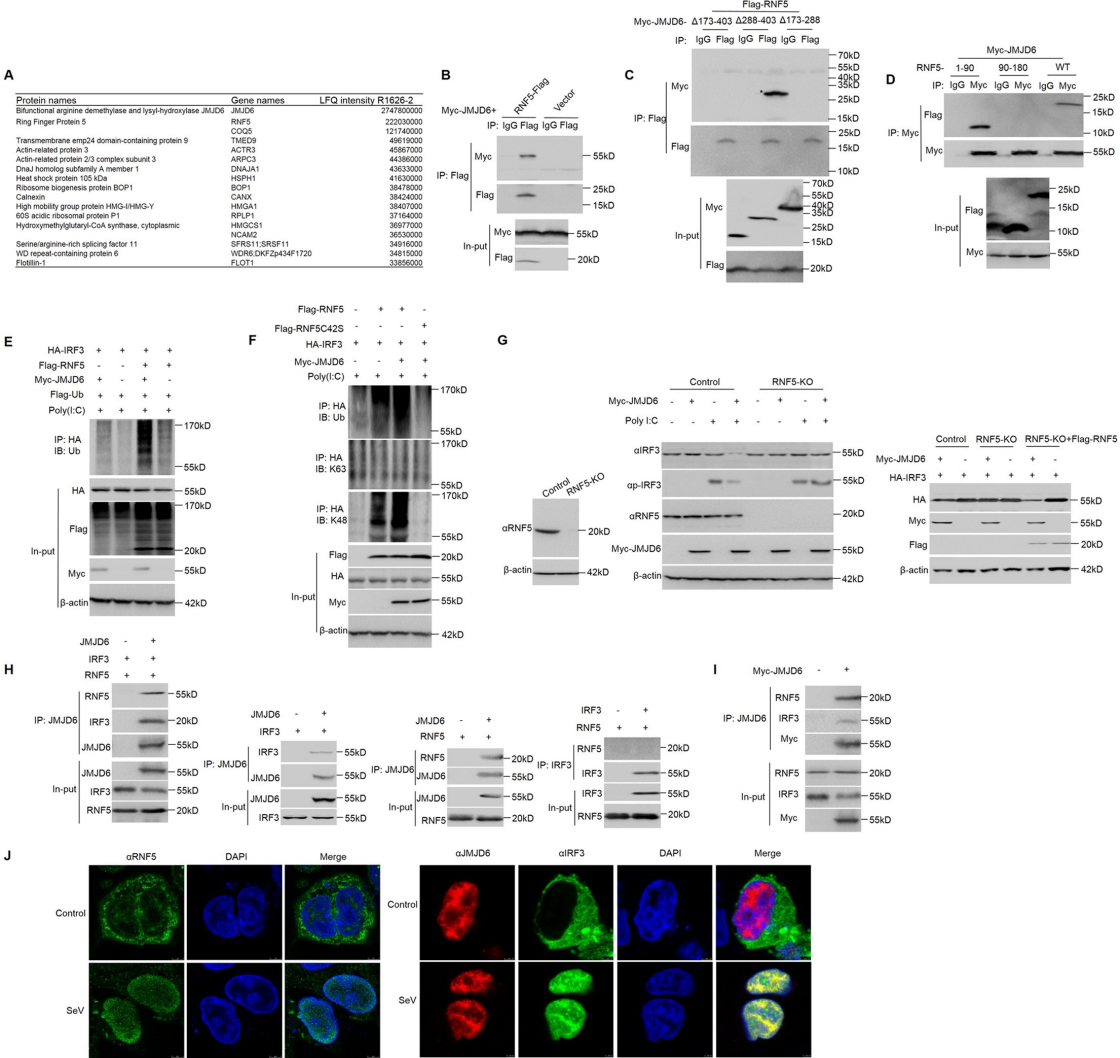
JMJD6 promotes activated IRF3 K48 ubiquitination and degradation via ubiquitin ligase RNF5. (A) List of JMJD6 interactome based on Label-free quantification intensity. (B) Immunoblot analysis (with anti-Myc or anti-Flag antibodies) of proteins immunoprecipitated (with control IgG or anti-Flag) from lysates of HEK293T cells transfected with the indicated plasmids. The expression of the transfected proteins was analyzed by immunoblot. (C) HEK293T cells were transfected with the indicated plasmids, followed by immunoprecipitation with an IgG or anti-Flag antibody and immunoblotting with anti-Flag or anti-Myc antibodies. (D) HEK293T cells were transfected with the indicated plasmids, followed by immunoprecipitation with an IgG or anti-Myc antibody and immunoblotting with anti-Flag or anti-Myc antibodies. (E) Immunoblot analysis (with anti-Ub) of proteins immunoprecipitated (with anti-HA) from lysates of HEK293T cells transfected for 24 h with various combinations of plasmids (upper panels) and stimulated with poly(I:C) in the presence of MG132. The expression of Flag-RNF5, Myc-JMJD6, or HA-IRF3 was determined by immunoblot with the indicated antibodies (lower panels). (F) Immunoblot analysis (with anti-ubiquitin or antibody to K63-linked or K48-linked polyubiquitin (K63-ub or K48-ub, respectively); top) of proteins immunoprecipitated (with anti-HA) from lysates of HEK293T cells transfected for 24 h with various combinations of plasmids (upper panels) and stimulated with poly(I:C) in the presence of MG132. The expression of Flag-RNF5, Flag-RNF5C42S, Myc-JMJD6, or HA-IRF3 was determined by immunoblot with the indicated antibodies (lower panels). (G) Immunoblot analysis of RNF5 KO efficiency in HEK293T cells (Left panel). Immunoblot analysis of RNF5 KO cells transfected with Myc-JMJD6 or vector and secondarily transfected with poly(I:C), analyzed with anti-IRF3, anti-RNF5, and anti-Myc antibodies (Middle panel). The rescue experiment by ectopically expressing full-length of RNF5 in RNF5-knockout cells (Right panel). (H) In vitro interaction analysis of JMJD6 with RNF5 or IRF3 using in vitro-translated JMJD6, RNF5, and IRF3. JMJD6, RNF5, and IRF3 were obtained by in vitro transcription and translation. Far-left panel, the interaction between JMJD6 and RNF5 or IRF3 was assayed by mixing JMJD6 and RNF5 or IRF3, followed by IP with JMJD6 antibody. Left panel, the interaction between JMJD6 and IRF3, followed by IP with JMJD6 antibody. Right panel, the interaction between JMJD6 and RNF5, followed by IP with JMJD6 antibody. Far-right panel, the interaction between IRF3 and RNF5, followed by IP with IRF3 antibody. (I) Immunoblot analysis of both IRF3 and RNF5 in a JMJD6-immunoprecipitated complex upon virus infection. HEK293T cells were transfected for 24 h with or without Myc-JMJD6 in the presence of SeV infection. (J) Fluorescent images of HEK293T cells stimulated with or without SeV (MOI of 1) for 12 h. DAPI-stained nuclei are shown in blue. Left panel, RNF5 was detected with green fluorescence. Right panel, JMJD6 was detected with red fluorescence, and IRF3 was detected with green fluorescence.

To gain insights into the E3 ligase recruited by JMJD6 to degrade activated IRF3, we characterized the JMJD6 interactome using affinity purification and mass spectrometry (S3 Fig). Among JMJD6-associated proteins, ring finger protein 5 (RNF5) is an E3 ubiquitin ligase (Fig 6A). To confirm the interaction of JMJD6 with RNF5 protein, we examined whether JMJD6 binds to RNF5 by IP-western analysis after overexpressing Flag-RNF5 or control vector for 24 h in transiently transfected HEK293T cells. As shown in Fig 6B, ectopically expressed JMJD6 was detected in the RNF5 immunoprecipitation. RNF5 binds to a sequence within the intermediate domain of JMJD6 (amino acids 173–288 of JMJD6), but not the C-terminal or N-terminal region of JMJD6 (Fig 6C). We further confirmed JMJD6 binds to a sequence with N terminal 90 residues (RING domain) of RNF5, but not the C-terminal region of RNF5 (Fig 6D). We found JMJD6 associated with both activated IRF3 and RNF5, and IRF3 polyubiquitylation in cells was increased in the presence of RNF5 and JMJD6 (Fig 6E). RNF5C42S, in which the Cys42 in the ring-finger domain is mutated to serine, is well accepted as an inactive catalytic mutant of RNF5 [23]. As shown in Fig 6F, overexpression IRF3 exhibited little ubiquitylation of IRF3, but it was actively ubiquitylated by a K48-linked polyubiquitin chain upon expression of both JMJD6 and RNF5. On the other hand, RNF5C42S abolished the K48-linked polyubiquitin chain to ubiquitylate activated IRF3 (Fig 6F). Ubiquitination is an enzymatic post-translational modification in which a ubiquitin-protein is attached to a substrate protein. This process most commonly binds the last amino acid of ubiquitin to a lysine residue on the substrate. The ubiquitination tags the protein for degradation via the proteasome pathway. The lysine 48 (K48)-linked ubiquitin chain is one of the most abundant chains and a major proteasome-targeting signal in cells [24]. K48-linked ubiquitination often leads to the degradation of target proteins by the 26S proteasome; consistent with the above observations, proteasome inhibitor MG132 abrogated JMJD6-induced degradation of IRF3. And K48-specific antibody recognized ubiquitin signaling of proteins immunoprecipitated with IRF3 from lysates of HEK293T cells transfected for 24 h with various combinations of plasmids and stimulated with poly(I:C) in the presence of MG132 (Fig 6F). All of these indicate JMJD6 leads to degradation of K48 ubiquitination of activated IRF3 in an RNF5-dependent fashion.

To further confirm if JMJD6 promoted activated IRF3 ubiquitylation via RNF5-based E3 complex, we generated RNF5 KO HEK293T cells using the CRISPR-Cas9 system (Fig 6G). In KO control cells, JMJD6 dramatically reduced activated IRF3 and p-IRF3 expression. However, JMJD6 failed to reduce activated IRF3 and p-IRF3 expression in the RNF5 knockout (KO) cells. To confirm the effect of RNF5 toward JMJD6-mediated activated IRF3 degradation and excluded the off-target effects associated with the CRISPR-Cas9 system, we performed a rescue experiment by ectopically expressing full-length of RNF5 in RNF5-knockout cells. Expression of the full-length of RNF5 in RNF5-knockout cells resulted in complete restoration of JMJD6-mediated activated IRF3 level reduction (Fig 6G). In addition, protein-protein interactions *in vitro* were performed to confirm the interaction among JMJD6, RNF5, and IRF3. RNF5, IRF3, and JMJD6 were generated in an *in vitro* protein expression system. Co-immunoprecipitated with anti-JMJD6 in a mixture of these proteins showed a direct interaction between JMJD6 and IRF3 or RNF5, but RNF5 couldn’t bind to IRF3 (Fig 6H). Meanwhile, both IRF3 and RNF5 could be detected in the JMJD6-immunoprecipitated complex upon SeV infection (Fig 6I). All of these indicate JMJD6, RNF5, and IRF3 compose a complex. HEK293T cells were infected with or without SeV, the subcellular localization of RNF5, IRF3, or JMJD6 was investigated. As shown in Fig 6J, RNF5 and IRF3 showed significant translocation from the cytoplasm to the nucleus upon SeV infection, and JMJD6 remains in the nucleus upon SeV infection, indicating IRF3 was degraded in the nucleus upon SeV infection. These results showed that JMJD6 recruited RNF5 to promote activated IRF3 K48 ubiquitination in the nucleus upon SeV infection.

### JMJD6 deficient mice are more resistant to VSV infection

**Fig 7:**
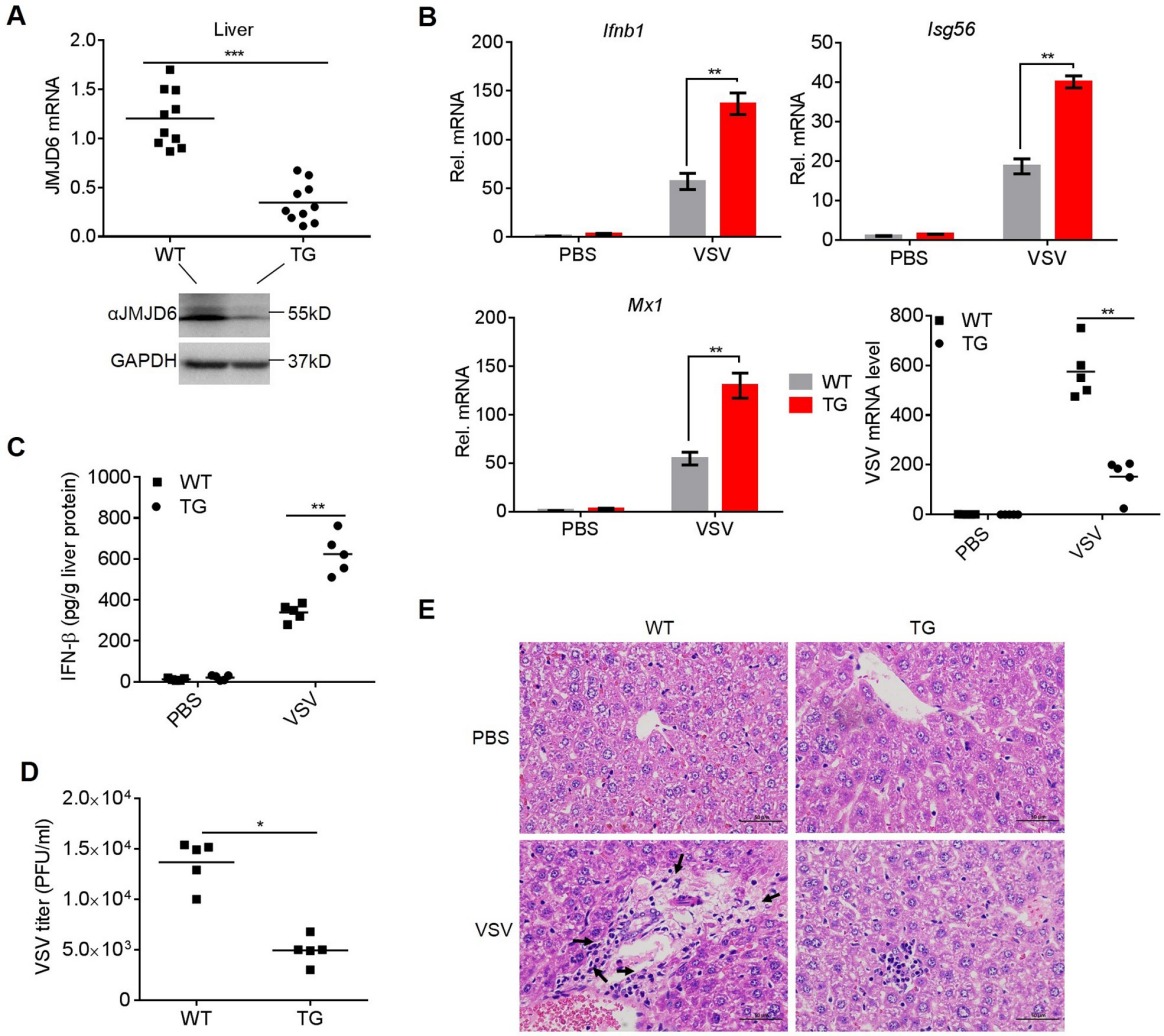
JMJD6 deficient mice are more resistant to VSV infection. (A) The knockdown efficiency of TG in mouse livers was detected by qPCR (top) and Western blotting (below). (B) qPCR analyses of the levels of the Ifnb1, Isg56, and Mx1 mRNAs and VSV load in the livers of WT and TG mice injected intraperitoneally with PBS or VSV for 48 h. (C) ELISA analysis of IFN-β production in the liver from WT and TG mice intravenously infected with VSV for 48 h. The lowercase letter "g" is the symbol for the gram. (D) The VSV titers of liver from WT and TG mice were analyzed by the standard plaque-forming unit assay. (E) Images of H&E staining of livers sections from the mice. Inflammatory cells are indicated by a black arrowhead.

To assess the physiologic relevance of this study, we evaluated the importance of JMJD6 in antiviral host defense *in vivo*. Loss-of-functions studies have shown JMJD6 is essential in embryogenesis and tissue differentiation. JMJD6 KO mice die neonatally [25]. Therefore, we employed the piggyBac transposon system by hydrodynamic injection via the tail vein to knockdown JMJD6 expression in mice liver (TG). Consequently, piggyBac transposon was able to achieve efficient JMJD6 knockdown in the liver (Fig 7A). Wildtype (WT) and TG mice were challenged with VSV by tail vein injection. VSV load, ISGs expression, and histopathology in the liver of mice were monitored. As shown in Fig 7B, TG mice had increased IFN-β, Isg56, and Mx1 mRNA expression in the liver 48 h post-injection (Fig 7B). TG mice also had reduced VSV mRNA levels in the liver at 48h post-injection (Fig 7B).

Moreover, the amount of IFN-β protein induced by VSV infection was much higher in TG mice’s liver than in VSV-infected WT mice (Fig 7C). Reverse correlative with enhanced IFN-β production, VSV titers in the liver were significantly reduced in TG mice than in WT mice (Fig 7D). HE staining showed that reduced infiltration of inflammatory cells was observed in the liver after VSV injection compared with WT mice (Fig 7E). Thus, these data indicate JMJD6 deficiency protects mice against VSV infection.

## Discussion

Studies have identified functions for JMJD6 in a variety of biological processes, including cell differentiation, proliferation, migration, and apoptosis [26, 27]. Increased expression of JMJD6 has frequently been detected in many different neoplasms [28]. Here we have demonstrated that JMJD6 inhibits IRF3-dependent transcriptional activation, thereby providing negative regulation of the innate antiviral responses. The results presented here indicate that increased expression of JMJD6 suppresses the innate antiviral responses and promotes viral replication, which may partly explain why cancer cells are more susceptible to viral infection since abundant JMJD6 expression in cancer cells [29, 30].

We have demonstrated that JMJD6 suppresses IFN-I production via degradation activated IRF3. Since constitutively active IRF3 is harmful to cells [31], IRF3 activation and deactivation must be strictly regulated. Our results here have demonstrated that JMJD6 negatively regulates activated IRF3 to control the activated IRF3. JMJD6 interacted with IRF3 after poly(I:C) stimulation, which induces the phosphorylation and activation of IRF3. In contrast, the treatment of cell lysates with λ-PPase, which could dephosphorylate IRF3, abolished the poly(I:C)-induced interaction between JMJD6 and IRF3 (Fig 4C), suggesting that JMJD6 binds to IRF3 in a phosphorylation-dependent manner. It is worth noting that JMJD6 degraded activated IRF3 through the ubiquitin-dependent proteasome pathway. Phosphorylation of proteins is functionally coupled with protein degradation via the ubiquitin-dependent proteasome pathway [32]. However, JMJD6 is not an E3 ubiquitin ligase; therefore, the additional E3 ubiquitin ligase should be involved in the process. Several lines of evidence showed that RNF5, an E3 ubiquitin ligase, was involved in JMJD6-mediated degradation of activated IRF3. Firstly, it specifically interacted with JMJD6 via the RING domains (Fig 6D). Secondly, RNF5 markedly increased JMJD6-mediated ubiquitination of IRF3 (Fig 6E). Thirdly, the substitution of the conserved cysteine residue in the RING domain of RNF5 abolished its E3 ligase activity in IRF3 ubiquitination (Fig 6F). Fourthly, KO of RNF5 abolished the JMJD6-induced degradation and ubiquitination of IRF3 (Fig 6G). These data indicate that RNF5 was responsible for JMJD6-mediated degradation of IRF3. Moreover, RNF5 and IRF3 showed significant translocation from the cytoplasm into the nucleus upon viral infection (Fig 6J), which indicates that JMJD6 recruits RNF5 and IRF3 together in the nucleus upon viral infection. Of note, RNF5 doesn’t interact with IRF3 directly in vitro binding assays (Fig 6H). These indicate that JMJD6 catches RNF5 to degrades IRF3 in the nucleus upon viral infection.

RNF5 has been reported in ER stress, autophagy, and cancers [33–35]. Recent studies have identified several RNF5 substrates as central participants in innate immune responses, such as MITA and MAVS, involving innate immune signaling pathways [36, 37]. Here, we showed IRF3 as another substrate of RNF5, which expands the biological functions of RNF5 in immune regulation. It has been reported that the E3 ubiquitin ligases were recruited by intermediate protein to degrade the substrate [38]. For example, Smad2 recruits E3 ligase Smurf2 to degrade SnoN [39]. Numb recruits E3 ligase Itch to degrade Notch1 [40].

**Fig 8:**
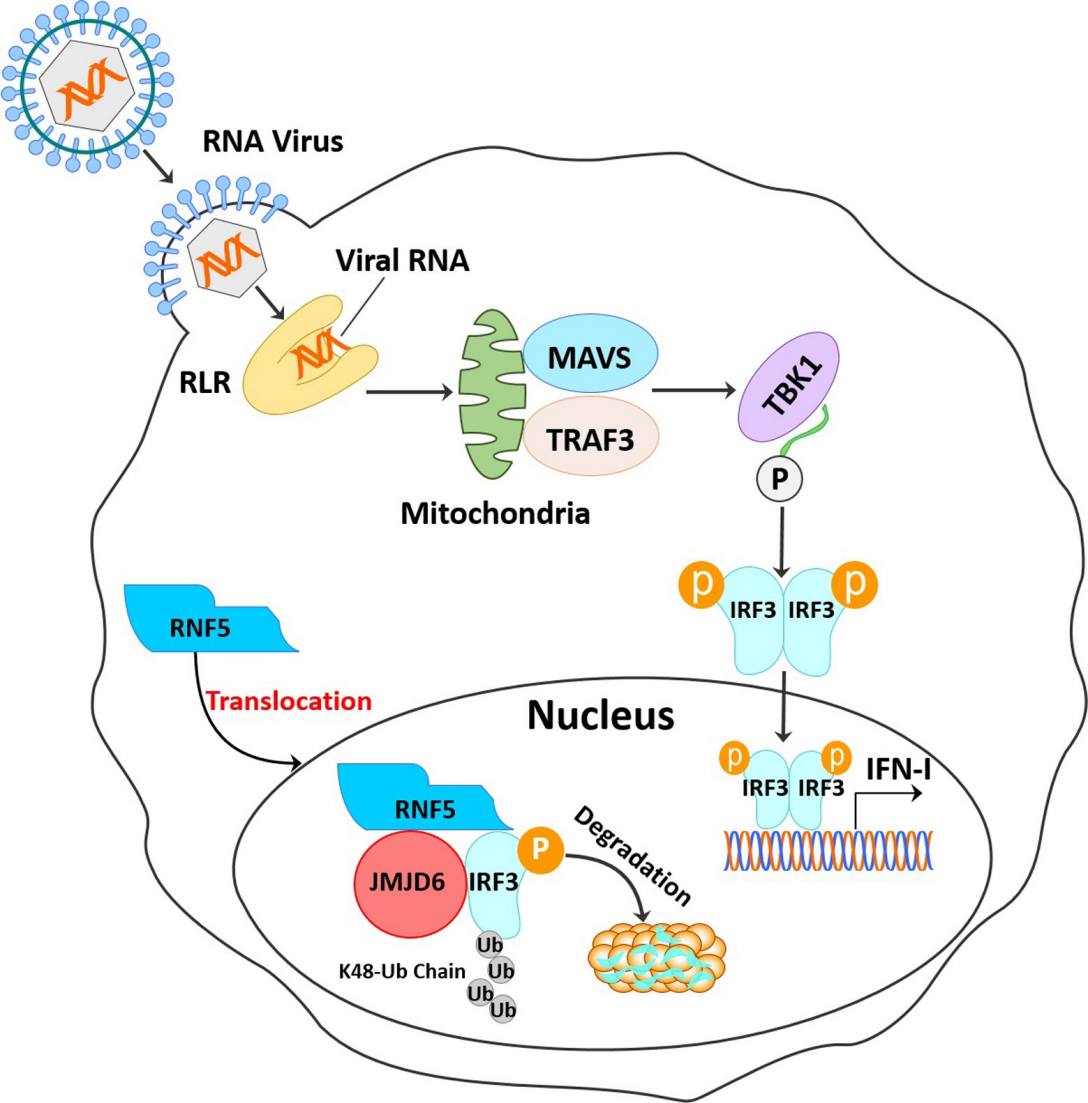
Schematic illustration of JMJD6 serving as a negative immune regulator by inhibiting IFN-I production. JMJD6 suppresses the RLR sensing pathway by degrading activated IRF3 in an RNF5-dependent manner.

In summary, we showed that JMJD6 is a negative regulator in IRF3-mediated innate immune signaling by recruited RNF5 to degrade activated IRF3 and promote viral replication *in vitro* and *in vivo* (Fig 8). Understanding the role of JMJD6 in innate immunity may shed light on the natural protection occurring in RNA virus infection.

## Materials and methods

### Ethics statement

All animal experiments were approved and performed according to the requirements of the Gansu Animal Experiments Inspectorate and the Gansu Ethical Review Committee (Licence no. SYXK (GAN) 2010–003).

### Viruses and cells

VSV-GFP was kindly provided by Prof. Bo Zhong (Wuhan University, China) and amplified in Vero cells. Sendai virus (SeV) was kindly provided by Prof. Hongbing Shu (Wuhan University, China). SeV was amplified in SPF eggs as previously described [41]. Briefly, SPF eggs were bought from Taizhou, Shandong Province, and incubated in an incubator for 9 days. Each egg was inoculated with 100 μL of SeV virus solution (viral stock diluted with PBS at 1:100), and the allantoic fluid was collected after 72 h incubation. Human embryonic kidney 293T (HEK293T) cells and HeLa cells were cultured at 37°C under 5% CO_2_ in Dulbecco modified Eagle’s medium (DMEM, Gibco, USA) supplemented with 10% fetal bovine serum (FBS). RNF5-KO-HEK293T cells were provided by Qiyun Zhu (Lanzhou veterinary Research Institute).

### Reagents and antibodies

Lambda protein phosphatase (λPPase) was purchased from New England Biolabs (NEB, USA). The MG132 (20 μM) was purchased from Merck & Co (Germany). The chloroquine diphosphate (CQ) (100 μM), benzyloxycarbonyl (Cbz)-l-Val-Ala-Asp (OMe)-fluoromethylketone (Z-VAD-FMK) (50 μM), and poly(I:C) (50 ng/ml) were purchased from Sigma-Aldrich (MI, USA). Antibodies against p-IRF3 (Cell Signaling Technology (CST), Cat #4947), IRF3 (CST, Cat #4302), p-TBK1 (CST, Cat #5483), TBK1 (CST, Cat #3013), Ubiquitin (CST, Cat #3936), Ubiquitin K63-specific linkage (CST, Cat #5621), Ubiquitin K48-specific linkage (CST, Cat #8081), HA (Biolegend, Cat #901513), Flag (Santa Cruz Biotechnology, Cat #sc-166355), Myc (Santa Cruz Biotechnology, Cat #sc-47694), and β-actin (Santa Cruz Biotechnology, Cat #sc-47778), JMJD6 (Abcom, Cat #ab176172 and Proteintech, Cat #16476-1-AP), and GAPDH (Proteintech, Cat #60004-1-Ig) were purchased from the indicated manufacturers.

### Plasmids

ISRE, Nifty, and IFN-β promoter-luciferase reporter plasmids, mammalian expression plasmids for HA-IRF3, IRF7, MAVS, MDA5, RIG-I, and TBK1, were generously donated by Hongbing Shu (Wuhan University). The pcDNA3.1-Myc-JMJD6, JD6-Δ173–408, JD6-Δ288–408, and JD6-Δ173–288 constructs have been described [41]. In brief, the pcDNA3.1-Myc-JMJD6, JD6-Δ173–408, JD6-Δ288–408, and JD6-Δ173–288 plasmids were generated through inserting the fragment into a pcDNA3.1/myc-His A vector (Invitrogen, Carlsbad, CA, USA). The PCR primer pairs are shown in S1 Table. All constructed plasmids were analyzed and verified by DNA sequencing.

### Transfection and reporter gene assays

HEK293T cells were seeded on 24-well plates at a density of about 1×10^6^ cells per well overnight before transfection. Cells were then co-transfected with 100 ng of a reporter plasmid such as pIFN-β-Luc or pISRE-Luc and 200 ng of expression plasmid as indicated by lipofectamine 2000 transfection reagent (Invitrogen). To normalize transfection efficiency, 20 ng of the renilla luciferase reporter plasmid pRL-TK was added to each transfection. At 24 h post-transfection, cells were infected with SeV for 12 h, and luciferase assays were performed with a dual-specificity luciferase assay kit (Promega). Data are represented as mean ± SEM. ***P<0.001, **P<0.01 and *P<0.05 determined by two-tailed Student’s *t*-test.

### Co-immunoprecipitation and Western blotting analysis

HEK293T cells were cultured in 10-cm dishes, and the monolayer cells were co-transfected with various plasmids. The collected cells were then lysed and immunoprecipitated. For Western blotting, target proteins were resolved by SDS-PAGE and transferred onto an Immobilon-P membrane (Millipore). The membrane was blocked and incubated with appropriate primary antibodies and secondary antibodies. The antibody-antigen complexes were visualized using enhanced chemiluminescence detection reagents (Thermo).

### RNA extraction and qPCR

Total RNAs were extracted using TRIzol Reagent (Invitrogen). The cDNA was synthesized from the extracted RNA samples, using the M-MLV reverse transcriptase (Promega) and random hexamer primers (TaKaRa). The Mx3005P QPCR system (Agilent Technologies) and SYBR Premix ExTaq reagents (TaKaRa) were used in the quantitative PCR (qPCR) experiment to quantify the abundance of various mRNAs. Primers used for qPCR assays were listed in S2 Table. All target gene expression was normalized to the control gene encoding GAPDH in each sample, and the 2^-ΔΔCt^ method was used to calculate relative expression changes.

### RNA interference (RNAi)

Small interfering RNA (siRNA) used in the RNAi assay was chemically synthesized by GenePharma (China). The knockdown of endogenous JMJD6 in HEK293T cells was carried out using transfection of JMJD6 siRNA. Nontargeting siRNA (NC siRNA) was used as a negative control. The transfection of siRNA was performed using Lipofectamine 2000. The target sequence for human JMJD6 was 5’-CUGGCCACCUGAAUUCAAATT-3’.

### CRISPR-Cas9 knockout

To generate JMJD6 KO cells, double-stranded oligonucleotides corresponding to the target sequences were cloned into the lenti-CRISPR-V2 vector, and then lentiviral particles were harvested and used to transduce HeLa cells. The infected cells were selected with puromycin (1 μg/mL) for 2 weeks before additional experiments were carried out. The sgRNA sequence used in this study was designed using the online CRISPR design tool (http://crispr.mit.edu/), and the sequence was 5’- ATCAAAGTGACCCGAGACGA-3’. The genomic region surrounding the CRISPR target site was amplified by PCR using the check primers (forward, 5’-CCATTCTGTAGGTGGTTTGTGA-3’; reverse, 5’-TTGTAAGATTTCCAGGGGTTTG-3’), and the PCR products were purified and sequenced.

### Generation of a JMJD6 stable overexpressing cell line

The JMJD6 gene was cloned into the pLV-ISRE-Puro vector (Clontech). For packaging lentiviruses, 3 μg of pLV-ISRE-Puro-JMJD6 plasmid, 2 μg of psPAX2 packaging plasmid (Addgene, 12260), and 1 μg of pMD2.G envelope plasmid (Addgene, 12259) were co-transfected into HEK293T cells (4×10^6^). The supernatants containing lentiviruses were collected, filtered, and stored at -80°C. For infection, HEK293T cells were incubated with viral stocks for 24 h and then supplied with a fresh medium. Cells were selected with 1 μg/ml puromycin (Gibco, A1113803) at 24 h post-infection.

### *In vitro* lambda protein phosphatase treatment

According to the manufacturer’s instruction, cell lysates were subjected to lambda PPase (New England Biolabs) treatment. Control reactions lacking the phosphatase were performed in parallel. The assay mixture was then used to perform the co-IP assay, as described above.

### PiggyBac knockdown transgenic (TG) mice

Cas9 and gRNA were gifts from Professor Sen Wu’s lab as a piggyBac transposon (PB) vector. Then PB with specific JMJD6 gRNA was mixed with PBase at a ratio of 3:1 and then injected via mouse tail vein as the published protocol [42]. Each group contains 5 male C57BL/6J mice at the age of six weeks. Mice were injected with 12 μg PiggyBac transposon vector with gRNA and 4 μg plasmid expressing piggyBac transposase. 5×10^7^ PFU VSV per mouse was used for viral infection. As a mock control, mice were injected with 12 μg PiggyBac transposon vector lacking gRNAs and 4 μg plasmid expressing piggyBac transposase.

### ELISA assay

Cell supernatants from infected or uninfected cells were collected at different time points post SeV infection. Qualitative detection of human IFN-β was performed by ELISA according to the manufacturer’s instructions (Elabscience, Cat#E-EL-H0085c). The quantification of mouse IFN-β in livers were measured by ELISA Kits (MLBio, Cat#ml063095) according to the manufacturer’s instructions. Briefly, livers were rinsed in ice-cold PBS to remove excess blood thoroughly and weighed before homogenization. Minced the livers to small pieces and homogenized them in cold PBS (tissue weight (g): PBS (mL) volume = 1:9). The resulting suspension was subjected to two freeze-thaw cycles to further break the cell membranes. After that, the homogenates were centrifugated for 15 minutes at 5000 rpm. Supernatants were then subjected to mouse IFN-β ELISA. Quantification of IFN-β was calculated according to the manufacturer’s instructions.

### Statistical analysis

All data are presented as mean ± SEM of at least three independent experiments. Statistical significance was determined with a two-tailed Student’s t-test (***P<0.001, **P<0.01, and *P<0.05).

## Supporting information

S1 Fig JMJD6 deficiency potentiates the type I interferon production. Alignment of the JMJD6 genomic nucleotide sequence of the published JMJD6 reference sequence and the KO-control, KO-JMJD6-1, and KO-JMJD6-2 sequences using LaserGene software. The red box indicates the regions that were mutated (top panel). Confirmation of the genome editing by Sanger sequencing the PCR amplicon from the JMJD6 genome of the cell lines (below panel).
(TIF)

S2 Fig JMJD6 promotes activated IRF3 degradation. (A) Exogenous JMJD6 regulated the stabilities of IRF3. Immunoblot of lysates from HEK293T cells transiently expressing HA-IRF3 and Myc-JMJD6 or Myc-JD6-Δ173–288 stimulated with poly(I:C) and then cultured in the presence of CHX. (B) Exogenous JMJD6 enhanced the ubiquitination of IRF3. Immunoblot analysis (with anti-Ub) of proteins immunoprecipitated (with anti-HA) from lysates of HEK293T cells transfected for 36 h with HA-IRF3 and Myc-JMJD6 or Myc-JD6-Δ173–288 and stimulated with poly(I:C) in the presence of MG132 (20 μM).
(TIF)

S3 Fig JMJD6 mediates degradation of activated IRF3 via the ubiquitin ligase RNF5. HEK293T cells were transfected with a plasmid expressing vector or Myc-JMJD6, and the cell lysates were immunoprecipitated with anti-Myc and then resolved by SDS-PAGE and silver-stained.
(TIF)

S1 Table The PCR primer pairs used in this study. (XLSX)

S2 Table Primers for mRNA Quantification. (XLSX)
