# Supplementary figures and images for: JMJD6 negatively regulates cytosolic RNA induced antiviral signaling by recruiting RNF5 to promote activated IRF3 K48 ubiquitination
Source: PLoS Pathog. 2021 Mar 8;17(3):e1009366. doi: 10.1371/journal.ppat.1009366 (PMC7971890; doi:10.1371/journal.ppat.1009366)

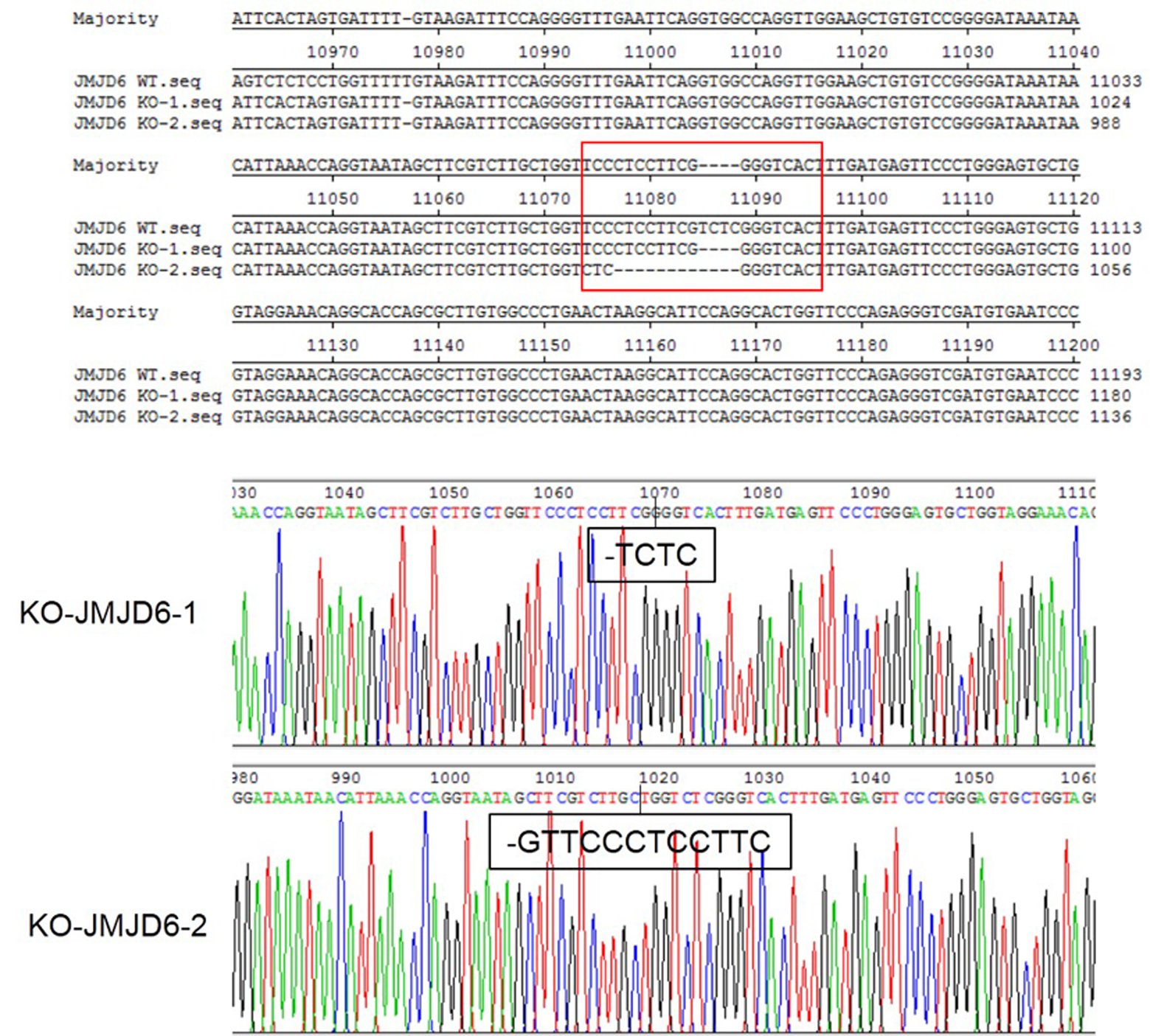

Supplement: S1 Fig — Alignment of the JMJD6 genomic nucleotide sequence of the published JMJD6 reference sequence and the KO-control, KO-JMJD6-1, and KO-JMJD6-2 sequences using LaserGene software. The red box indicates the regions that were mutated (top panel). Confirmation of the genome editing by Sanger sequencing the PCR amplicon from the JMJD6 genome of the cell lines (below panel). (TIF) [file ppat.1009366.s001.tif]

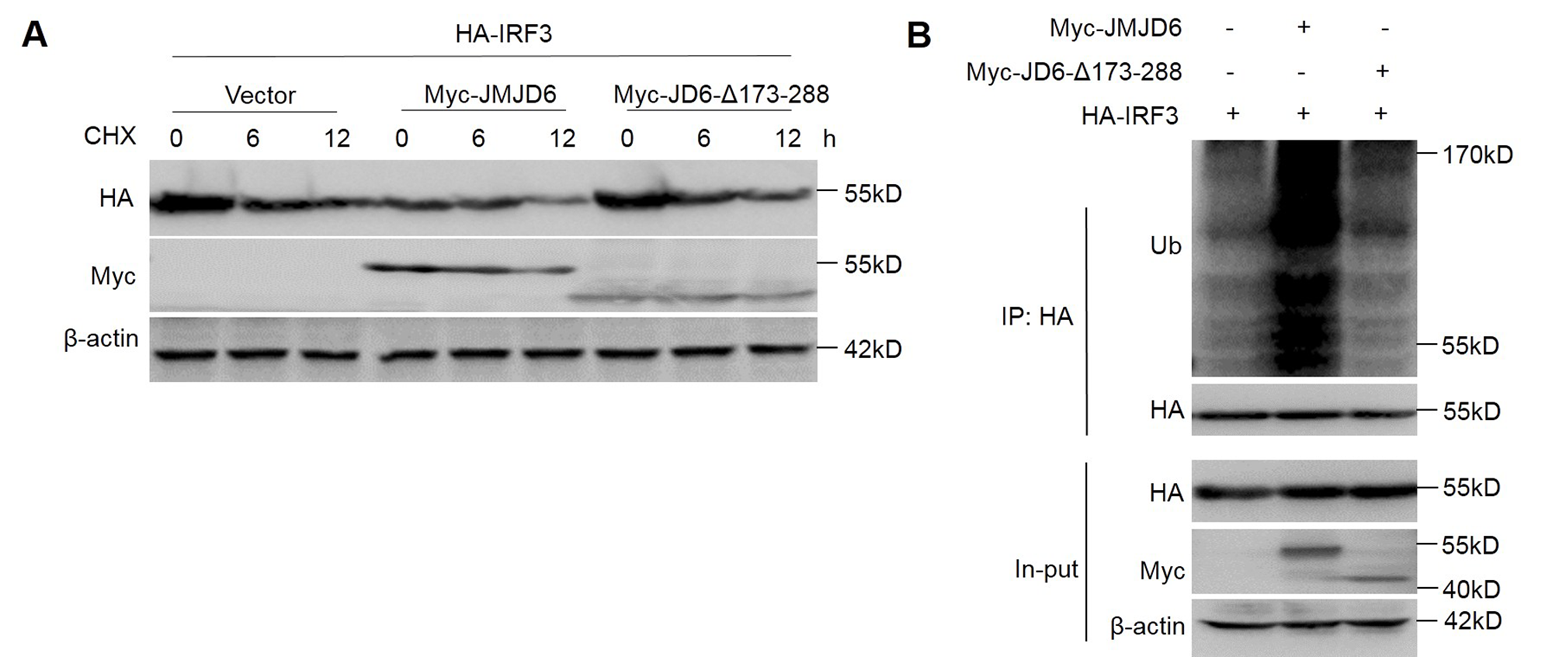

Supplement: S2 Fig — (A) Exogenous JMJD6 regulated the stabilities of IRF3. Immunoblot of lysates from HEK293T cells transiently expressing HA-IRF3 and Myc-JMJD6 or Myc-JD6-Δ173–288 stimulated with poly(I:C) and then cultured in the presence of CHX. (B) Exogenous JMJD6 enhanced the ubiquitination of IRF3. Immunoblot analysis (with anti-Ub) of proteins immunoprecipitated (with anti-HA) from lysates of HEK293T cells transfected for 36 h with HA-IRF3 and Myc-JMJD6 or Myc-JD6-Δ173–288 and stimulated with poly(I:C) in the presence of MG132 (20 μM). (TIF) [file ppat.1009366.s002.tif]

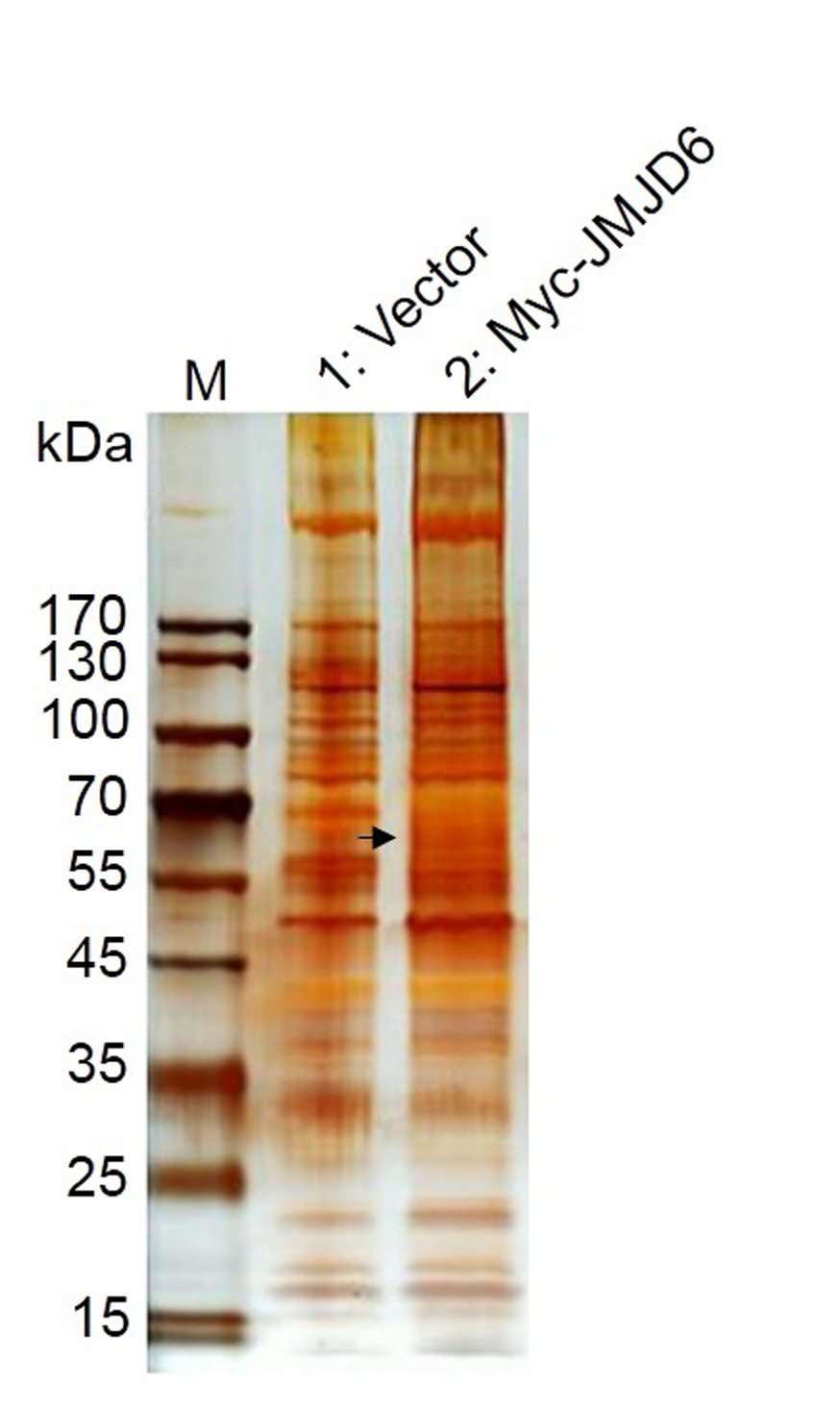

Supplement: S3 Fig — HEK293T cells were transfected with a plasmid expressing vector or Myc-JMJD6, and the cell lysates were immunoprecipitated with anti-Myc and then resolved by SDS-PAGE and silver-stained. (TIF) [file ppat.1009366.s003.tif]
